# Recent Advances in Liquid Biopsy of Brain Cancers

**DOI:** 10.3389/fgene.2021.720270

**Published:** 2021-09-17

**Authors:** Yunyun An, Fei Fan, Xiaobing Jiang, Kun Sun

**Affiliations:** ^1^Shenzhen Bay Laboratory, Shenzhen, China; ^2^Department of Neurosurgery, Union Hospital, Tongji Medical College, Huazhong University of Science and Technology, Wuhan, China; ^3^BGI-Shenzhen, Shenzhen, China

**Keywords:** peripheral blood, cerebrospinal fluid, cell-free DNA, noninvasive, cancer diagnosis

## Abstract

Brain cancers are among the top causes of death worldwide. Although, the survival rates vary widely depending on the type of the tumor, early diagnosis could generally benefit in better prognosis outcomes of the brain cancer patients. Conventionally, neuroimaging and biopsy are the most widely used approaches in diagnosis, subtyping, and prognosis monitoring of brain cancers, while emerging liquid biopsy assays using peripheral blood or cerebrospinal fluid have demonstrated many favorable characteristics in this task, especially due to their minimally invasive and easiness in sampling nature. Here, we review the recent studies in the liquid biopsy of brain cancers. We discuss the methodologies and performances of various assays on diagnosis, tumor subtyping, relapse prediction as well as prognosis monitoring in brain cancers, which approaches have made a big step toward clinical benefits of brain cancer patients.

## Introduction

Brain malignancies are big threats to public health worldwide. It is estimated that brain cancer accounts for ~1.3% of newly diagnosed cancer patients and~3.0% of deaths in the United States; in China, researchers estimated that for brain cancer, each year there are more than 100,000 new cases along with 60,000 deaths (Chen et al., 2016; Siegel et al., 2021; Sung et al., 2021). Based on the histological criteria and genotypes, brain cancers could be classified into more than 150 subtypes, including various kinds of primary and secondary/metastatic tumors (Louis et al., 2016). The most common primary brain cancer is known as glioblastoma (GBM); while metastatic tumors account for a much larger proportion in brain cancers, and the tumors could originate from various tissue sources including the lungs, breast, kidney, colon, and skin (Bos et al., 2009; Kircher et al., 2016; Nozawa et al., 2017; Yousefi et al., 2017; Yekeduz et al., 2020). The stage, subtype, as well as somatic mutation landscapes, could dominate the therapeutic strategies for the best benefits of brain cancer patients. In clinical, brain tumors growing in functional areas are usually diagnosed at relatively early stages due to significant symptoms, while the majority of brain tumors are not easy to be diagnosed which leads to the unsatisfactory prognosis of the patients (Lapointe et al., 2018). Hence, early diagnosis and molecular subtyping of tumors are both of high clinical value for better healthcare of brain cancer patients.

Conventional diagnosis and biopsy of brain tumors rely on advanced imaging and histopathological techniques. In imaging, there are three widely used methods: computed tomography (CT), positron emission tomography (PET), and gadolinium-enhanced MRI. Due to the high resolution and sensitivity, MRI is the most popular method for brain cancer detection (Villanueva-Meyer et al., 2017; Wadhwa et al., 2019); CT usually serves as an alternative imaging modality for patients with metallic implants and embedded devices (Maroldi et al., 2005; Pope, 2018); PET uptakes target-specific radiotracers to profile the metabolism and functional changes in the brain, which may occur early than morphological changes (Jones et al., 2012; Suchorska et al., 2014). Compared to traditional tracers in the PET utility, such as ^11^C-methionine and ^18^F-fluoro-deoxyglucose, emerging radiolabeled amino acid tracers and their analogs (e.g., ^18^F-fluoroethyltyrosine, and ^18^F-fluorodopa) show better performance in detecting glioma extent without additional technical enhancement in PET imaging (Garnett et al., 1983; Suchorska et al., 2014; Verger et al., 2017; Katsanos et al., 2019; Treglia et al., 2019). Currently, to get precise and multi-aspect information, integrated applications of multiple tools are frequently used, such as PET/CT and PET/MRI (Treglia et al., 2014, 2019). However, neuroimaging with improper interpretations may lead to untimely or excessive therapeutic responses (Peca et al., 2009; Neagu et al., 2015; Shankar et al., 2017). Consequently, novel approaches for brain cancer diagnosis have been developed in recent years, and liquid biopsy is one of them.

Liquid biopsy uses body fluids [e.g., peripheral blood, and cerebrospinal fluid (CSF)] as the working material and utilizes various analytes, such as circulating tumor DNA (ctDNA), and proteins. Liquid biopsy is considered minimally invasive and can provide key genetic and epigenetic information of the tumor. Currently, liquid biopsy has been implemented in various clinical scenarios, including non-invasive diagnosis of brain cancer, subtyping the brain tumor, prognosis monitoring and prediction of minimal residue diseases and prognosis, as well as many other applications to improve the treatment benefits of the patients (Peng et al., 2017; Wan et al., 2017).

## Peripheral Blood and Cerebrospinal Fluid

**Figure 1:**
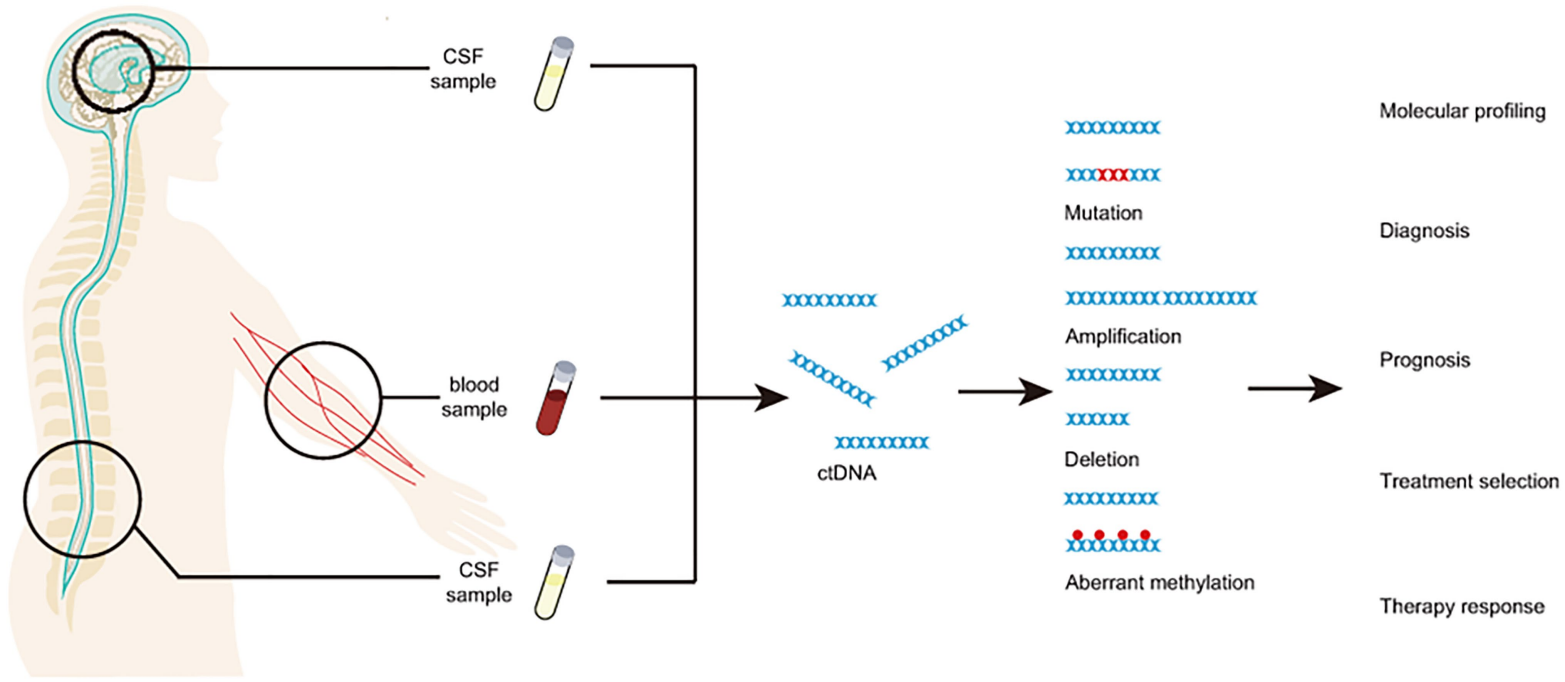
CfDNA from blood and/or CSF as a minimally invasive analyte for liquid biopsy of brain cancer.

In brain cancer diagnosis, peripheral blood and CSF are the most popular materials (Figure 1 and Table 1; Siravegna et al., 2017b; Fontanilles et al., 2018; Yan et al., 2021). Circulating tumor DNA isolated from peripheral blood is effective for diagnosis and subtyping of brain cancer. For example, the detection of copy number abbreviation and promoter methylation (e.g., MGMT and PTEN) from ctDNA of serum by Methylation specific PCR (MSP) can help with the diagnosis of astrocytomas and oligodendrogliomas of various grades with high specificity (while sensitivity may not be that optimal; Lavon et al., 2010). Similar results are also found by another study on promoter methylation profiles by MSP of MGMT, RASSF1A, p15INK4B, and p14ARF in serum (Majchrzak-Celinska et al., 2013). In fact, hypermethylation of various genes can be easily detected in patients with primary or metastatic central nervous system (CNS) cancer (Majchrzak-Celinska et al., 2013). One optimally verified score matric, the “glioma-epigenetic liquid biopsy score” or GeLB can help with distinguishing glioma patients with 100% sensitivity and 97.78% specificity (Sabedot et al., 2021). Using the 5hmC-Seal technique, scientists identify healthy individuals from patients with WHO II-III gliomas and GBM and not be affected by glioma-related pathological features, such as Isocitrate Dehydrogenase [NADP (+)] 1 (IDH1) mutation (Cai et al., 2021). Similar to IDH1 mutation, 5hmC can be a proper biomarker for distinguishing patients with GBM from gliomas, indicating the potential utility of 5hmC in gliomas screening (Cai et al., 2021). Another biomarker, Telomerase Reverse Transcriptase (TERT), has been validated for diagnosis in gliomas patients, with overall 62.5% sensitivity and 90% specificity (Muralidharan et al., 2021). With the development of next-generation sequencing (NGS), studies show that about half of patients (211 out of 419) with GBM or other primary brain tumors have detectable ctDNA, indicating high potential in clinical utilities (Piccioni et al., 2019). Besides peripheral blood, CSF is another widely used material for liquid biopsy of brain cancers. In fact, various ctDNA characteristics, including promoter methylation and mutation profiles, are highly consistent in the peripheral blood and paired CSF from the same patients as demonstrated in a comparative study (Liu et al., 2010). For instance, Ma et al. (2020) show that mutated EGFR gene can be detected in CSF in 81.8% non-small cell lung cancer patients with leptomeningeal metastases. Another example is that Histone 3 p.K27M (H3K27M) mutation, could be detected in 88% patients with diffuse midline glioma (DMG) from both CSF and plasma and the changes of H3K27M agreed with 83% tumor response to radiotherapy (Panditharatna et al., 2018). The utility of droplet digital PCR (ddPCR) makes H3K27M a powerful biomarker in detecting pediatric DMG and monitoring therapy responses (Panditharatna et al., 2018). A study for gliomas patients by detecting of IDH1, TERT, and H3K27M also showed high detection sensitivity (71%, 20 out of 28) and specificity (Fujioka et al., 2021). Very recently, with the detection of somatic copy number alterations and the analysis of DNA fragmentation patterns, scientists can identify glioma patients using untargeted and low-coverage whole-genome sequencing technique, which makes diagnosis and subtyping cheaper and more time-saving (Mouliere et al., 2018). Notably, a recent study had integrated multiple cfDNA fragmentation patterns to build a machine learning classifier on urine samples, which shows promising accuracy in differentiating glioma patients from negative controls (Mouliere et al., 2021). Besides early diagnosis and subtyping, liquid biopsy assays have also been developed for disease progression monitoring and therapeutic methods assessments of brain cancer patients. For instance, Murtaza et al. (2015) demonstrated the utility of truncal gene mutations of ctDNA in multifocal clonal evolution monitoring of the tumors in a breast cancer patient with brain metastasis. Blood-based genomic sequencing of liquid biopsy in the primary treatment of ALK-positive non-small cell lung cancer patients with CNS metastases indicated the superior efficacy of alectinib than chemotherapy, suggesting the functions of liquid biopsy in evaluating therapeutic effects (Zhu et al., 2020b).

**Table 1:** Recent studies in brain cancer diagnosis.

Source	Related diseases	Biomarkers	Information provided and findings	Approaches	Clinical applicability	References
Blood	Glioblastoma (GBM)	MGMT	Promoter methylation	PCR, Methylation specific PCR (MSP)	Therapy response	Lavon et al., 2010
Blood	GBM	IDH1, EGFR, TP53, PTEN	Mutations	Next-generation sequencing (NGS)	Molecular profiling/diagnosis	Bettegowda et al., 2014
Blood	GBM	IDH1	Mutations	Droplet digital PCR (ddPCR)	Diagnosis	Boisselier et al., 2012
Blood	GBM	Genomic variants	Genomic variants	NGS	Diagnosis	Nabavizadeh et al., 2020
Blood	GBM and other primary brain tumors	ERBB2, MET, EGFR	Mutations, amplifications	NGS	Molecular profiling	Piccioni et al., 2019
Blood	GBM, astrocytoma, gliosarcoma, meningiomas and metastatic CNS cancer	MGMT, RASSF1A, p15INK4B, and p14ARF	Promoter methylation	MSP	Diagnosis	Majchrzak-Celinska et al., 2013
Blood	Metastatic CNS cancer (breast cancer)	ERBB2	Mutations	ddPCR	Diagnosis	Garcia-Murillas et al., 2019
Blood	Metastatic CNS cancer (breast cancer)	Multiple genes	Mutations	WES	Diagnosis/therapy response	Murtaza et al., 2015
Blood	Metastatic CNS cancer (lung cancer)	ARID1A, STAT3,TP53,BRCA1,CTNNB1,EML4-ALK	Mutation	NGS	Molecular profiling/diagnosis/therapy response	Zhu et al., 2020b
Blood	Metastatic CNS cancer (melanoma)	BRAF, NRAS and c-KIT	Mutations	ddPCR	Diagnosis/therapy response	Lee et al., 2020
Blood	Gliomas	Telomerase reverse transcriptase (TERT)	Mutations	ddPCR	Diagnosis	Muralidharan et al., 2021
Blood	Gliomas	DNA methylation	Methylation	NGS	Diagnosis/therapy response	Sabedot et al., 2021
Blood	Gliomas, GBM	5hmC	Methylation	NGS	Diagnosis	Cai et al., 2021
Cerebrospinal fluid (CSF)	GBM	R132H, H3F3A	Mutations	WES	Diagnosis	Duan et al., 2020
CSF	Primary brain tumors (PBTs)	BRAF V600E	Mutations	BNA-PCR clamping	Diagnosis	Nakano et al., 2020
CSF	Gliomas	EGFR, PTEN	Somatic copy number alterations	NGS	Diagnosis	Mouliere et al., 2018
CSF	GBM	IDH1, TERT, and H3K27M	Mutations	ddPCR	Molecular profiling/diagnosis	Fujioka et al., 2021
Blood and CSF	GBM	MGMT, p16INK4a, TIMP3, THBS1	Promoter hypermethylation	MeDIP and real-time PCR	Diagnosis	Liu et al., 2010
Blood and CSF	Metastatic CNS cancer (breast cancer)	TP53, PIK3CA, ERBB2 and cMYC	Mutations, amplifications	ddPCR, WES	Diagnosis/prognosis	Siravegna et al., 2017a
Blood and CSF	Metastatic CNS cancer (lung cancer)	EGFR	Mutations	ddPCR	Diagnosis	Huang et al., 2019
Blood and CSF	GBM, Medullo and metastatic CNS cancer	IDH1, TP53, PTEN, EGFR, FGFR2, ERBB2	Mutations	ddPCR	Diagnosis/prognosis	De Mattos-Arruda et al., 2015
Blood and CSF	Brainstem gliomas	H3F3A, TP53, ATRX, PDGFRA, FAT1, HIST1H3B, PPM1D, IDH1, NF1, PIK3CA and ACVR1	Mutations	NGS	Diagnosis	Pan et al., 2019
Blood and CSF	Medullo	TP53,TNNB1,KMT2D,MYC,PRDM6,PTEN,SUFU	Mutations, amplification, insertion, deletion, gain	WES	Diagnosis/prognosis/treatment selection/therapy response	Escudero et al., 2020
Blood and CSF	Metastatic CNS cancer (lung cancer)	EGFR, KIT, PIK3CA, TP53, SMAD4, ATM, SMARCB1, PTEN, FLT3, GNAS, STK11, MET, CTNNB1, APC, FBXW7, ERBB4, and KDR	Mutations	NGS	Diagnosis	Ma et al., 2020
Blood and CSF	Diffuse midline glioma (DMG)	H3K27M	Mutations	ddPCR	Diagnosis/therapy response	Panditharatna et al., 2018
Blood and CSF	DMG	H3.3K27M	Mutations	ddPCR	Diagnosis	Li et al., 2021
Blood and CSF	Gliomas	TERT, IDH1, TP53, PTEN and other mutated genes	Mutations and fragmentation patterns	NGS	Diagnosis	Mouliere et al., 2021

*BNA, bridged nucleic acid; CNS, central nervous system; DMG, diffuse midline glioma; ddPCR, droplet digital PCR; GBM: glioblastoma; MeDIP, methylated DNA immunoprecipitation; Medullo, medulloblastoma; MSP, methylation specific PCR; NGS, next-generation sequencing; and WES, whole exome sequencing*.

On the other hand, although, the specificities of most blood-based liquid biopsies are relatively high, the sensitivities vary a lot. Sensitivities of these assays depend on multiple factors, including cancer types, tumor volumes, tumor vessel sizes, immune cell density, and other morphological characteristics. One study shows that ctDNA in plasma carrying tumor-specific mutations could only be detected in only 10% glioma, 50% neuroblastoma, and 60% medulloblastoma patients (Bettegowda et al., 2014). Other studies show that the IDH1^R132H^ mutation detection rate increases with largening tumor volumes in glioma patients (Boisselier et al., 2012), while tumor vessel sizes and perivascular CD68 + macrophage density both affect the concentrations of ctDNA in plasma (Nabavizadeh et al., 2020). Several studies have indicated that the blood–brain barrier may inhibit the release of tumor cells or tumor cell products (e.g., ctDNA) into the bloodstream. For example, BRAF, NRAS, and c-KIT mutations can be detected in the plasma of melanoma patients with extracranial metastasis (64%), but not in patients with intracranial metastasis (Lee et al., 2020). Considering the limitations of peripheral blood, another body fluid, CSF, is much closer to brain tissues physically and may reflect tumor burden more directly (De Mattos-Arruda et al., 2015). Circulating tumor DNA concentration is usually higher in CSF compared to plasma, which means variations or mutations of low frequency are more likely to be detected in the CSF, therefore makes CSF-based liquid biopsy more powerful in the clinic (Russo et al., 2021). For instance, one study shows that in HER2-positive breast cancer patients with brain metastases, gene variations have higher levels in CSF than plasma for post-treatment monitoring (Siravegna et al., 2017a). Among patients with brainstem gliomas, 97.3% of cases with detectable alterations in the primary tumors are identified in CSF and the detection rates of mutated fragments are much higher in CSF (100%) than in plasma (38%; Pan et al., 2019). Other groups also show that ctDNA is more abundant and contains more comprehensive information in CSF than that of plasma in patients with GBM, medulloblastoma, and metastatic brain cancers (De Mattos-Arruda et al., 2015; Escudero et al., 2020; Mouliere et al., 2021). The advantage of CSF may be of particular value in developing liquid biopsy assays for prognosis monitoring and cancer relapse prediction. However, the collection of CSF is not as convenient as peripheral blood and may cause adverse effects to the patients in certain clinical scenarios, therefore, one must take careful considerations (especially the conditions of the patients) before applying CSF-based assays in real clinical settings.

## Advances and Challenges of Liquid Biopsy Approaches

Although, remarkable progress has been achieved in various aspects of modern medicine, early diagnosis, and effective treatment of brain tumors are still challenging. Conventional diagnostic methods, neuroimaging, and histopathological inspections are expensive, complex, and could only provide limited information for therapeutic benefits. In contrast, liquid biopsy assays utilizing plasma or CSF can provide patient-specific genetic information of the tumors. However, the detection rates and specificity of liquid biopsy assays show high fluctuations and may not be able to meet clinical requirements (Shankar et al., 2017; Fontanilles et al., 2018; Saenz-Antonanzas et al., 2019). To this end, advances in biotechnologies, such as ddPCR and Massive Parallel Sequencing, may promise a feasible path toward higher sensitivity and efficiency of liquid biopsy assays (Oellerich et al., 2017; Postel et al., 2017). ddPCR and other microfluidic-based technologies allow the detection of extremely rare events, such as identifying 0.1% tumor-derived fragments carrying somatic mutations from a large amount of background DNA (Xu et al., 2019), therefore, they are suitable for screening of hotspot mutations, or mutations associated with specific drugs. A new standard of liquid biopsy in pediatric DMG using ddPCR has been established, with nearly 100% specificity and sensitivity for H3.3K27M detection in CSF and plasma (Li et al., 2021). On the other hand, as a minimally invasive method, liquid biopsy can be performed regularly for monitoring of the disease process, treatment effect as well as prediction of relapse.

At the same time, other challenges are hindering the utility of liquid biopsy in routine clinical practice. Due to the blood–brain barrier, the concentration of tumor-derived cfDNA in plasma is usually very low in brain cancer patients, which makes some plasma-based liquid biopsy assays almost impossible to apply in a certain proportion of patients (Martinez-Ricarte et al., 2018). To this end, recent studies reported that focused ultrasound treatment could serve as a safe and effective technique to increase the release of biomarkers for assisting the successful implementation of liquid biopsies (Zhu et al., 2020a). For CSF-based assays, the routine extraction approach, lumbar puncture, sometimes brings adverse side effects to the patients and could not be performed frequently (Seoane et al., 2019). Lastly, most of the current studies are based on relatively small patient cohorts, hence, comprehensive validation studies or clinical trials using large-scale patient cohorts is essential for determining the appropriate assays for various clinical settings.

## Discussion

Overall, liquid biopsy using peripheral blood and CSF for brain tumors is an effective and minimal-invasive approach for brain cancer diagnosis in the clinic. As an effective and minimal-invasive approach, liquid biopsy using peripheral blood and/or CSF is useful for cancer diagnosis in an informative way, which is especially valuable under certain clinical scenarios, such as recent pandemic, COVID-19 (Pisapia et al., 2021). Widely used ddPCR, NGS, and methylated DNA immunoprecipitation in liquid biopsy promote the detection of mutations, copy number variations, and aberrant methylation and also help with the subtyping of brain tumors. With the help of neuroimaging and biopsy, we can use liquid biopsy to profile and monitor the tumor progression at different stages in both genetics and morphology and then choose better treatment plans. Meanwhile, currently liquid biopsy also has some disadvantages, especially limited sensitivity in blood-based assays. Cerebrospinal fluid-based assays show higher sensitivity and specificity, but it requires lumbar puncturing, which leads to the limited utility of CSF. Nevertheless, based on the promising results in the current studies, we believe that with further performance improvements and large-scale validations, liquid biopsy will certainly shine on brain cancer diagnosis and therapeutics in the near future.

## Author Contributions

KS and XJ designed research. YA, FF, XJ, and KS wrote the paper. All authors contributed to the article and approved the submitted version.

## Funding

This work is supported by Guangdong Basic and Applied Basic Research Foundation (2019A1515110173), BGI-research (BGIRSZ2020007), and Shenzhen Bay Laboratory.

## Conflict of Interest

The authors declare that the research was conducted in the absence of any commercial or financial relationships that could be construed as a potential conflict of interest.

## Publisher’s Note

All claims expressed in this article are solely those of the authors and do not necessarily represent those of their affiliated organizations, or those of the publisher, the editors and the reviewers. Any product that may be evaluated in this article, or claim that may be made by its manufacturer, is not guaranteed or endorsed by the publisher.
